# Clinical Application of Cone-Beam Computed Tomography of the Rabbit Head: Part 1 – Normal Dentition

**DOI:** 10.3389/fvets.2016.00093

**Published:** 2016-10-17

**Authors:** G. G. Riggs, Boaz Arzi, Derek D. Cissell, David C. Hatcher, Philip H. Kass, Amy Zhen, Frank J. M. Verstraete

**Affiliations:** ^1^Dentistry and Oral Surgery Service, William Pritchard Veterinary Medical Teaching Hospital, School of Veterinary Medicine, University of California Davis, Davis, CA, USA; ^2^Department of Surgical and Radiological Sciences, School of Veterinary Medicine, University of California Davis, Davis, CA, USA; ^3^Diagnostic Digital Imaging Center, Sacramento, CA, USA; ^4^Department of Population Health and Reproduction, School of Veterinary Medicine, University of California Davis, Davis, CA, USA

**Keywords:** cone-beam computed tomography, CBCT, rabbit, *Oryctolagus cuniculus*, dentition, anatomy

## Abstract

Domestic rabbits (*Oryctolagus cuniculus*) are increasingly popular as household pets; therefore, veterinarians need to be familiar with the most common diseases afflicting rabbits including dental diseases. Diagnostic approaches for dental disease include gross oral examination, endoscopic oral examination, skull radiography, and computed tomography (CT). CT overcomes many limitations of standard radiography by permitting cross-sectional images of the rabbit head in multiple planes without superimposition of anatomic structures. Cone-beam CT (CBCT) is an oral and maxillofacial imaging modality that produces high-resolution images. The objective of this study was to describe and compare the normal anatomic features of the dentition and surrounding maxillofacial structures in healthy rabbits on CBCT and conventional CT. Ten New Zealand white rabbit cadaver heads were scanned using CBCT and conventional CT. Images were evaluated using Anatomage *Invivo* 5 software. The maxillofacial anatomy was labeled on CBCT images, and the mean lengths and widths of the teeth were determined. The visibility of relevant dental and anatomic features (pulp cavity, germinal center, tooth outline, periodontal ligament) were scored and compared between conventional CT and CBCT. The thinnest teeth were the maxillary second incisor teeth at 1.29 ± 0.26 mm and the maxillary third molar teeth at 1.04 ± 0.10 mm. In general, it was found that CBCT was superior to conventional CT when imaging the dentition. Importantly, the periodontal ligament was significantly (*P* < 0.01) more visible on CBCT than on conventional CT. Ability to see the periodontal ligament with such detail may allow earlier detection and treatment of periodontal disease in rabbits. This study is the first of its kind and shows the feasibility and yield of CBCT when evaluating the maxillofacial features and dentition in rabbits.

## Introduction

Domestic rabbits (*Oryctolagus cuniculus*) are commonly afflicted by dental disease. Dental disease can result from trauma, improper diet and wear of the teeth, metabolic influences, and congenital abnormalities such as maxillary brachygnathism (1–9). Specific features of their dentition make rabbits susceptible to tooth overgrowth and malocclusion. Rabbits have a wider maxilla than mandible (anisognathia) and masticate in a laterolateral motion, predisposing them to lingual points on the mandibular teeth and buccal points on the maxillary premolar and molar teeth (2, 10). Rabbits have hypsodont dentition, i.e., their teeth have a long anatomic crown with only a small portion of the crown clinically visible in the oral cavity (clinical crown) and a larger portion of the crown residing within the alveolar bone (reserve crown) (10). The maxillary and mandibular first incisor teeth have enamel on the labial surfaces, and the mandibular first incisor teeth occlude directly on the maxillary second incisor teeth, resulting in chisel-shaped first incisor teeth. In contrast, the premolar and molar teeth are lophodont and have transverse enamel ridges on the occlusal surface of the teeth allowing for efficient mastication of fibrous foods (10–13). Lastly, rabbit teeth are open-rooted (i.e., elodont) and have a germinal center at the apical region from which they continuously grow (2, 11, 12). The maxillary first incisor teeth and mandibular incisor teeth have been reported to grow at a rate of 2.0 and 2.4 mm per week, respectively (2, 14). Sufficient wear of the teeth during mastication is critical for the maintenance of proper occlusion (1, 2).

Imaging techniques are essential in the diagnosis and evaluation of dental disease, in combination with history and physical examination findings. In rabbits, intraoral dental radiography is not currently possible due to the small aperture of the oral cavity and length of the teeth. In general practice, sedation and skull radiography are utilized to attempt evaluation of the dentition (2, 10–12, 15, 16). However, the diagnostic yield of conventional skull radiography is poor due to superimposition of the skull and the dentition. Computed tomography (CT) overcomes many of the imaging limitations of standard radiography by permitting cross-sectional images of the rabbit head in multiple planes without superimposition of anatomic structures (13, 17, 18). The technique is suitable for early detection of small changes in bone and adjacent soft tissues of the head and provides images of dental anatomy and pathological changes (13, 18–20). The use of CT for the diagnosis of dental abnormalities in rabbits is becoming the standard of care (13, 21).

With conventional CT, the slice thickness and in-slice resolution are usually on the order of 0.5–1.0 mm or higher. When scanning small objects, such as rabbit skulls, visualization of some structures is limited by image spatial resolution (18, 19). The disadvantage of the relatively low spatial resolution of CT versus radiographs can be compensated for by the lack of superimposition, ability to view images in multiple planes, and superior contrast resolution (18, 22). Since some rabbit teeth may only be 1 mm in width, conventional CT may not provide sufficient spatial resolution for the detection of subtle dental pathology in rabbits.

Cone-beam CT (CBCT) was introduced into the European market in 1998 and to the United States in 2001 (23). With the advent of CBCT, advanced imaging of small exotic species (e.g., rabbits, guinea pigs, and chinchillas) and small companion animals (i.e., cats and small-breed dogs) has the potential to become revolutionized. Compared to conventional CT, the patient ionizing radiation dose levels can be reduced by as much one-sixth (24). Scan times for CBCT are typically only 5–40 s, depending on the unit and protocol settings, allowing for comparable or decreased time under anesthesia (23). In full resolution mode, a CBCT can create 0.15-mm slices, and CBCT voxels are often isotropic (i.e., *X, Y*, and *Z* dimensions are equal), whereas conventional CT voxels are usually anisotropic with the best resolution occurring in the transverse plane (23). With both conventional CT and CBCT, software can reconstruct the two-dimensional images into tridimensional (3D) models, multiple image planes, and panoramic views aiding in the diagnosis and treatment planning for structures with complex geometry (25). The best quality reconstructions are achieved from images with fine spatial resolution and isotropic voxels, and CBCT is capable of producing exceptional image reconstructions. In human medicine, CBCT technology is being applied primarily to diagnosis and treatment of dental and maxillofacial disorders (25–29).

The objective of this study was to describe the anatomic features of the dentition and surrounding maxillofacial structures in healthy rabbits by means of CBCT and conventional CT (a.k.a., fan beam CT). We hypothesize that CBCT will be superior to conventional CT when imaging the dentition and osseous maxillofacial structures of the rabbit. The first aim of the study was to label the maxillofacial features and dentition of the CBCT images as well as determine the normal length and widths of the teeth. The second aim was to objectively compare the visibility of the germinal centers, pulp cavities, and periodontal ligaments as imaged by CBCT and conventional CT images. The global aim of this study is to provide the baseline for introducing CBCT for use in rabbits and to demonstrate the feasibility and yield of this technology in evaluating lagomorph maxillofacial features and dentition.

## Materials and Methods

### Specimens

Ten adult New Zealand white rabbit cadaver heads possessing full dentition and not showing overt evidence of dental disease were used in this study. These cadaver heads were obtained from rabbits that were humanely euthanized for other study purposes.

### Hardware and Software

The GE Lightspeed 16 conventional CT scanner and a NewTom 5G CBCT scanner were used in this study. Transverse images were obtained at a slice thickness of 0.2 mm for the CBCT and 0.625 mm for the conventional CT. Transverse conventional CT images were reconstructed using a bone filter, and CBCT images were reconstructed using the default manufacturer’s algorithm. The CBCT images were acquired using a field of view (FOV) of 18 cm width by 16 cm length.

Anatomage *Invivo* 5 software was used to evaluate the images in different orientations and windows. Multiple software tools were used to evaluate the CT images including (1) “bone view” for visualization of the bone surfaces; (2) “volume rendering” allowing evaluation of the 3D structure of different tissues; (3) “tooth view” for optimal visualization of the 3D structure of the teeth; and (4) “panoramic view” to generate images similar to dental radiographs. Images were further adjusted using the crop, rotate, and multi-planar reformatting tools to provide the best possible visualization of each anatomic structure.

### Gross Evaluation and Anatomy

**Figure 1:**
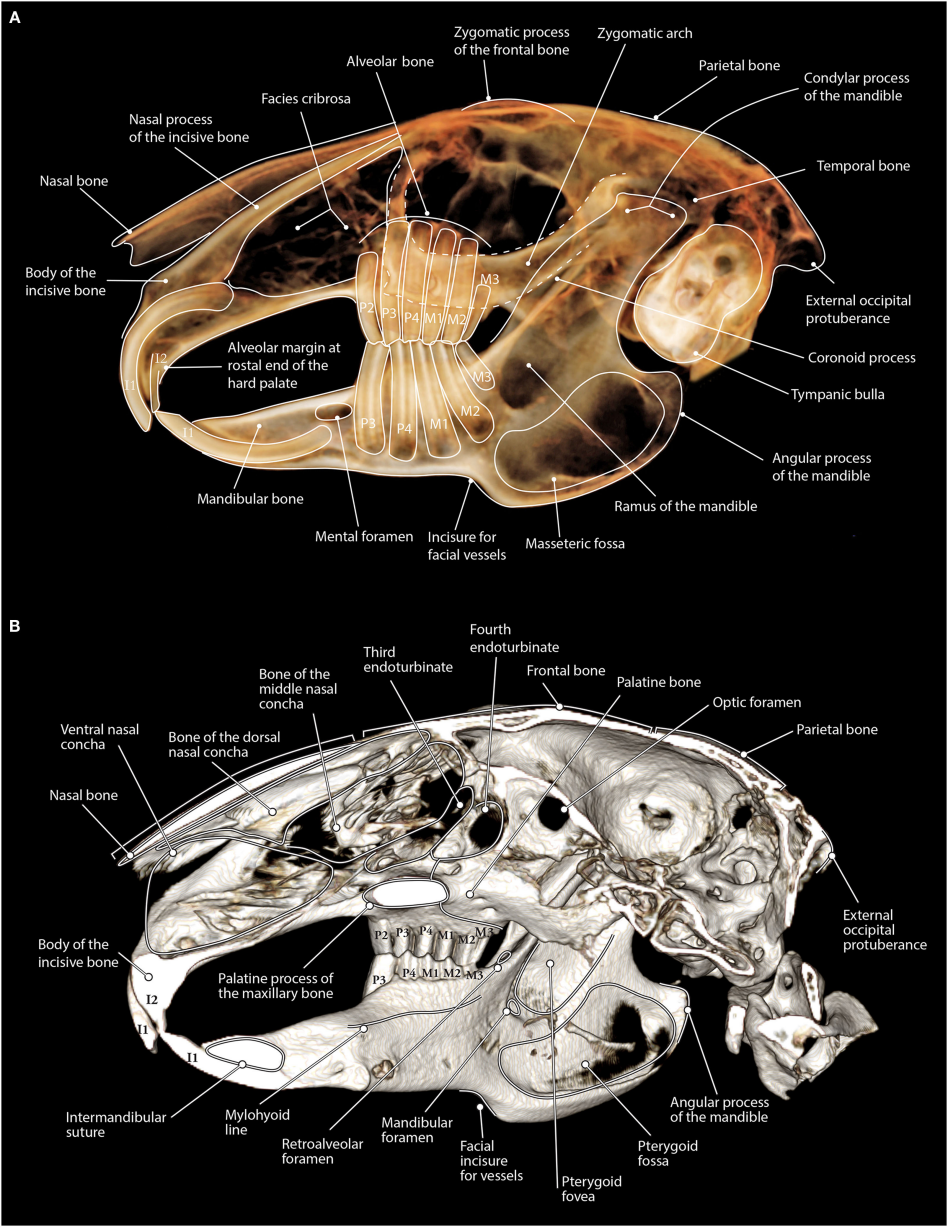
**(A)** Maxillofacial anatomy of a rabbit as viewed on tooth view 3D volume render (a non-shaded surface display) from CBCT data. The lateral aspect of the left side of the skull is displayed with the right side having been cropped from the image. Notice how the zygomatic arch is superimposed over the maxillary premolar and molar teeth. **(B)** Maxillofacial anatomy of a rabbit as viewed on bone mode 3D volume render (a shaded surface display) from CBCT data. The left half of the skull has been cropped so that the medial portion of the skull can be viewed. Notice the shape of the palatine process of the maxillary bone as this structure can be seen superimposed over the maxillary second, third, and fourth premolar teeth on panoramic images.

**Figure 2:**
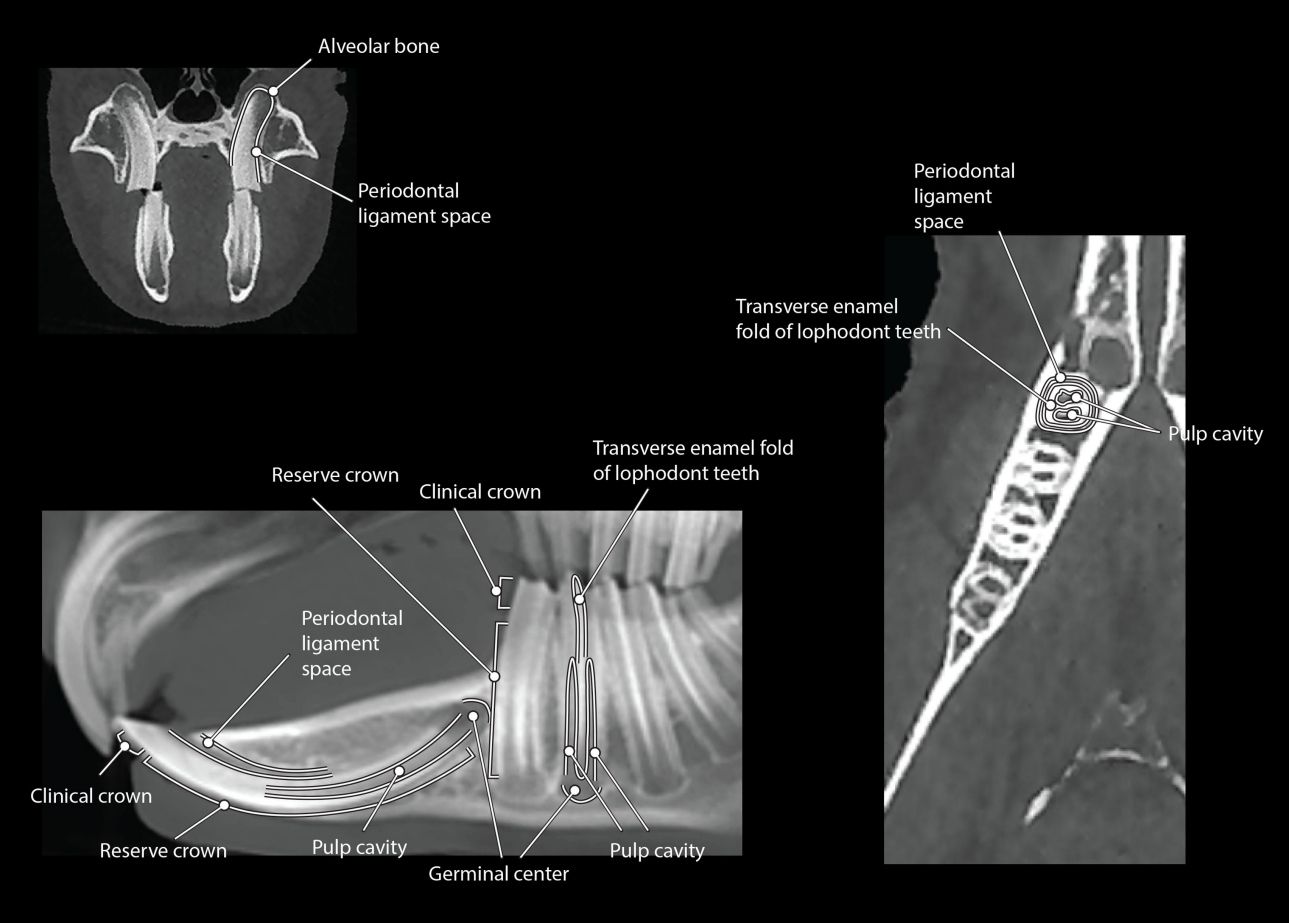
**The CBCT dental anatomy of a rabbit viewed in dorsal (right), transverse (top left), and MPR sagittal (bottom left) views within the section tab**.

The DICOM data from rabbit specimen #3’s CBCT were processed using the abovementioned software and evaluated on a medical-grade flat-screen monitor. 3D reconstructive, custom multi-planar reconstructed (MPR), dorsal, and transverse images were generated to assess and label the dentition and anatomy (Figures 1 and 2).

### Tooth Length and Width Measurements

**Figure 3:**
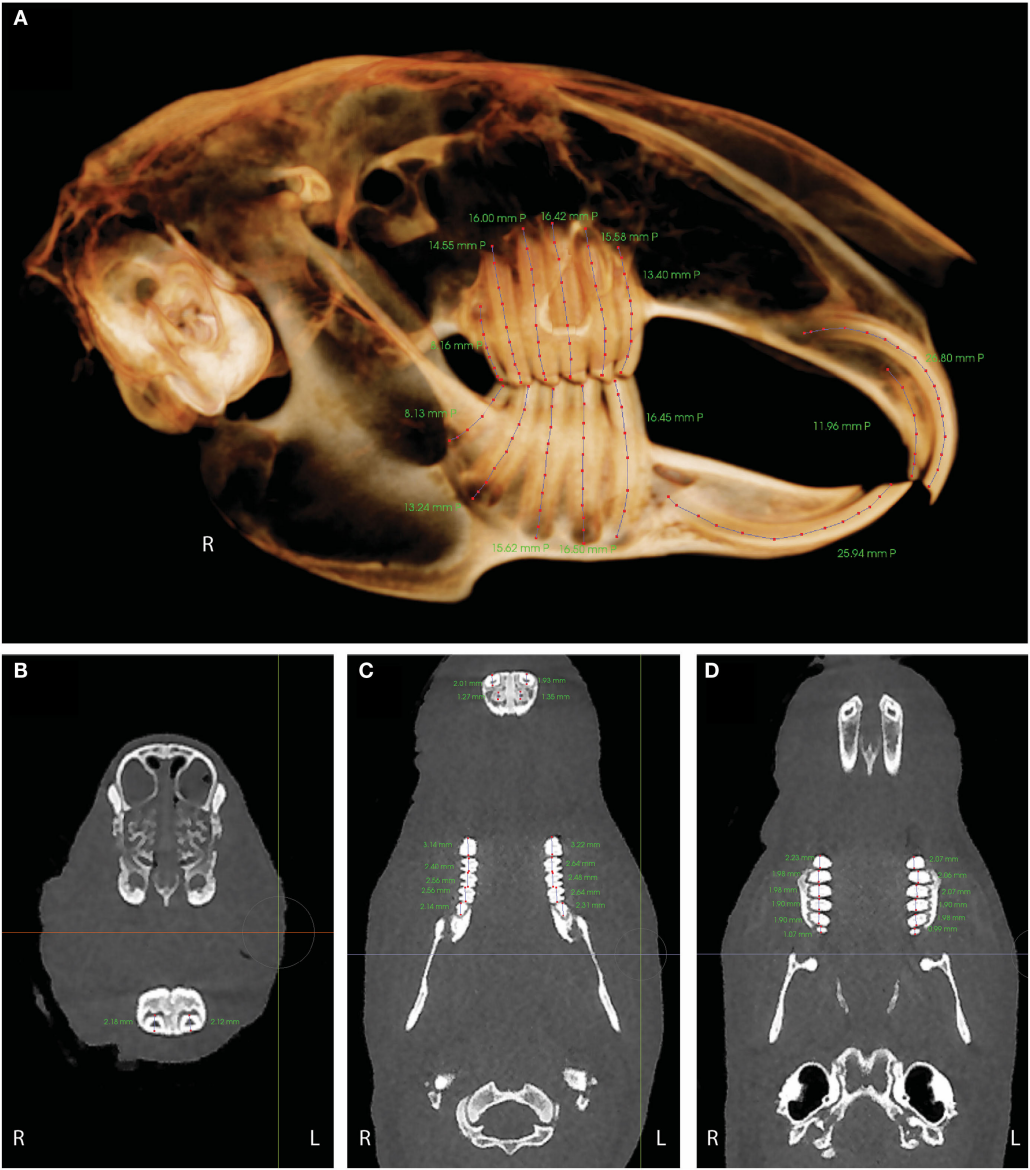
**(A)** Tooth length measurements in right lateral tooth view. mmP indicates the length in millimeters was performed on the two-dimensionally flattened volume rendered image. **(B)** The width of the mandibular incisor teeth was measured in a dorsoventral direction on a transverse image at the level of the teeth where they are the most ventral within the mandible. **(C)** Tooth widths for the mandibular premolar and molar teeth (middle of image) and the maxillary incisor teeth (top of image). **(D)** Tooth widths for the maxillary premolar and molar teeth. Tooth widths were evaluated in a rostral-to-caudal direction on a dorsal view image at the level of the alveolar margin.

Cone-beam CT skull images were set to be the same orientation. Using the “tooth view” volume rendering option, a sagittally oriented image was cropped to visualize the right or left side of the skull without summation of the contralateral side. The zygomatic arches were also cropped from the images to improve visualization of the maxillary premolar and molar teeth. Tooth measurements were performed on the two-dimensionally flattened volume rendered lateral views of the right and left hemisected skull images. A polygonal measuring tool was used to trace the central axis of each tooth, starting at the visible germinal center, following the curvature of the tooth, and ending at the visible crown (Figure 3A). The tooth length in millimeters was recorded.

Tooth widths for the maxillary and mandibular premolar and molar teeth and for the maxillary incisor teeth were evaluated in a rostral-to-caudal direction on a dorsal view image at the level of the alveolar margin (Figures 3C,D). The width of the mandibular incisor teeth was measured in a dorsoventral direction on a transverse image at the level of the teeth where they are the most ventral within the mandible (Figure 3B). The tooth width in millimeters was recorded.

### Sagittal and Multi-Planar Reconstruction Images and Scoring – CBCT and Conventional CT

**Figure 4:**
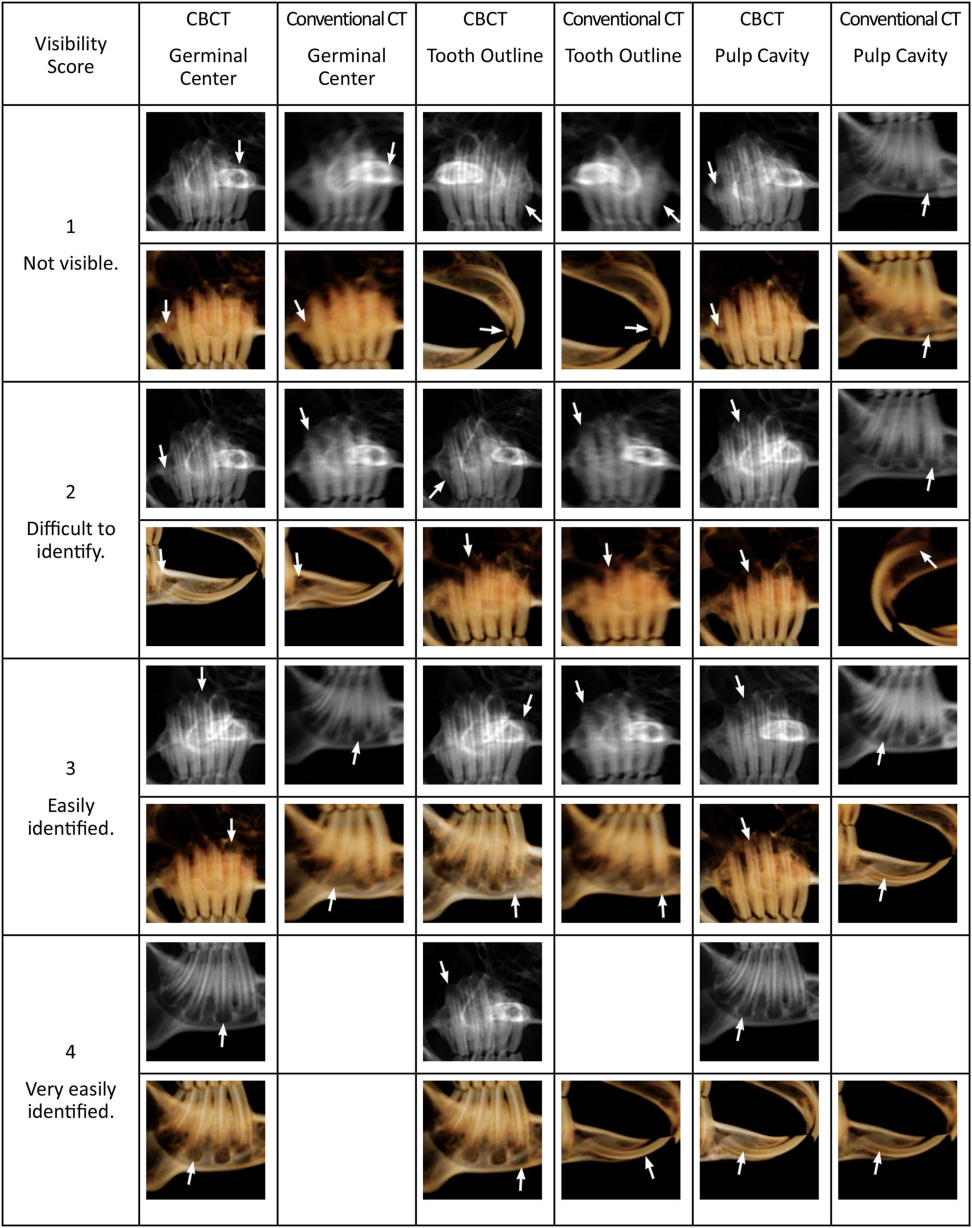
**Panoramic (grayscale) and tooth view (sepia) sagittal images from CBCT and conventional CT displaying the various scores for germinal center, pulp cavity, and tooth outline visibility**. The white arrows indicate the tooth and structure given the score. The zygomatic arch has been cropped from the images to facilitate viewing of the maxillary premolar and molar teeth. Blank boxes indicate that no teeth met these criteria.

**Figure 5:**
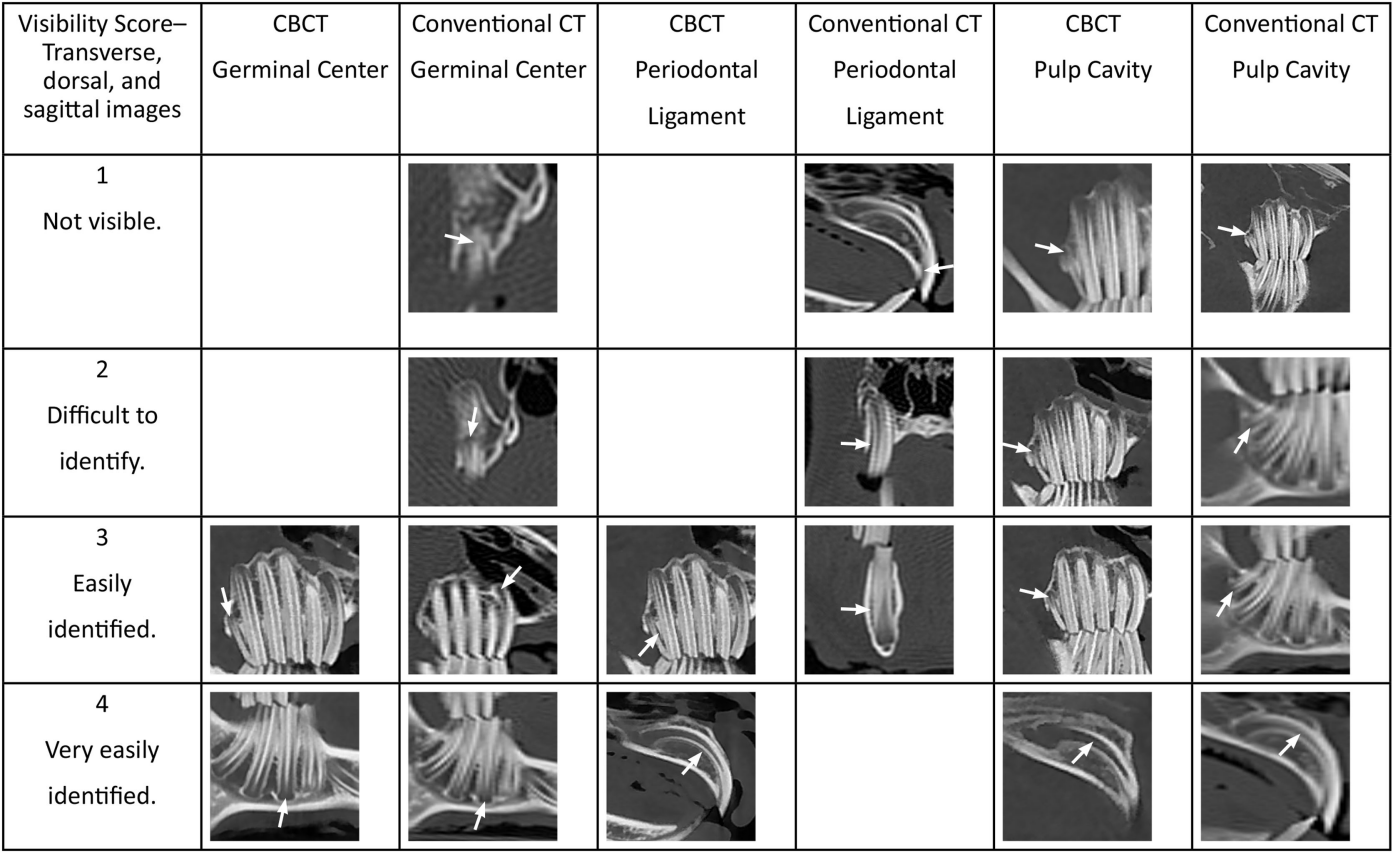
**The CBCT and conventional CT transverse, dorsal, sagittal, and custom MPR images displaying the various scores for germinal center, pulp cavity, and periodontal ligament visibility**. The white arrows indicate the tooth and structure given the score. Blank boxes indicate that no teeth met these criteria.

The zygomatic arch was cropped-out of the sagittal images, allowing better visualization of the maxillary premolar and molar teeth. Only one side of a hemisected skull was viewed at a time, to prevent superimposition of structures. Due to the obvious differences in image quality between the conventional CT and CBCT, the comparison of the images could not be blinded. The visibility of the germinal center, pulp cavity, and tooth outline for the sagittal images and the visibility of the germinal center, pulp cavity, and periodontal ligament for the MPR images were evaluated using an ordinal scoring system of 1–4 (Figures 4 and 5) using the tooth and panoramic views saved as TIF format images. For the germinal center and pulp cavity clarity on the sagittal and MPR images, a score of “1” was awarded to images in which the tooth was known to be present, but the structure was not identifiable or visible. For example, the germinal center region might be approximated, but the semicircle of the germinal center outline was not visible. Anatomic structures, such as the zygomatic arch, cropped portion of the zygomatic arch, mental foramen, mandibular foramen, and mandibular canal, may be completely blocking the structure from view. A score of “2” was awarded to images in which the structure was difficult to identify. For example, the germinal center region was identifiable, but the semicircle of the germinal center outline was inconsistent, obscured, and unclear. A radiolucent line may have been seen where the pulp cavity was known to be located, but the pulp cavity was indistinct, blurred, and pixelated. The image was not very useful in evaluating the pulp cavity for pathology. The same anatomic structures mentioned above may have partially obscured the structures. A score of “3” was awarded to images in which the germinal center and pulp cavity were easily identified. For example, the semicircle outline of the germinal center and the radiolucent dark triangle of the pulp cavity were mostly visible, but the image was not perfectly clear or crisp. There was very little overlap or interference of the other anatomic structures. The score of “4” was designated for images in which the structures were very easily identified. The semicircle outline of the germinal center and the radiolucent dark triangle of the pulp cavity were pronounced and visible in their entirety. The images were clear and crisp without any interference from the other anatomic structures.

The scoring system of 1–4 was applied to the tooth outline visibility for the sagittal images and to the periodontal ligament clarity for the MPR images. A score of “1” included teeth where <25% of the structure was distinguishable. The structure was indistinct, blurred, pixelated, or not identified at all. A score of “2” was awarded when 25–50% of the structure was distinguishable, but the remaining outline or periodontal ligament was indistinct, burred, or pixelated. A score of “3” was awarded when the structure was easily identified, but the image could be more clear and crisp. This included images where 50–75% of the structure was distinguishable. Finally, a score of “4” included images where 75–100% of the structure was distinguishable, pronounced, and visible in its entirety.

All 28 teeth were evaluated for the sagittal images; however, 5 representative teeth on the right side of the mouth were selected for evaluation on the transverse, dorsal, sagittal, and custom MPR images. The right maxillary first incisor tooth was selected for its large pulp cavity and arc shape. Visualization of the right maxillary second premolar tooth is obscured on sagittal images by the zygomatic arch and the palatine process of the maxilla; therefore, evaluation without these structures in the way would be beneficial. The right maxillary third molar tooth is the thinnest and shortest tooth in the mouth. The right mandibular fourth premolar tooth is oriented rather dorsoventral, allowing easy evaluation on transverse images. The right mandibular third molar tooth is the second shortest tooth and is oriented obliquely, making evaluation on purely transverse images challenging. The remaining teeth in the mouth have comparable features to these five teeth and therefore were not evaluated.

The paired scores from the CBCT versus conventional CT were compared using a Wilcoxon signed-rank test. A *P*-value <0.05 was considered significant. Statistical analysis was performed using statistics software (Stata IC/13.1, StataCorp LP, College Station, TX, USA).

## Results

### Gross Evaluation and Anatomy

The maxillofacial anatomy was easily identified on the bone and tooth mode 3D reconstruction images from the CBCT data (Figure 1). The dental anatomy was identified on the dorsal, custom sagittal MPR, and transverse images (Figure 2).

Despite optimal image manipulation using the contrast and opacity settings, the 3D reconstructed rabbit skull in bone mode appears to have fenestrations in the bone where the bone is very thin, such as in the masseteric fossa or the bony orbit, whereas the fenestrations in the bone at the facies cribrosa are anatomically normal. Occlusion is easily visualized in bone mode; however, care should be taken when evaluating the relationship of the mandible to the maxilla, as rabbits masticate in laterolateral movements, and the mandible may be shifted laterally due to patient position at the time of scanning despite normal occlusion.

The maxillofacial anatomy was also identifiable on the tooth mode reconstructions. Anatomic structures, such as the zygomatic arch, mental foramen, mandibular foramen, mandibular canal, and the palatine process of the maxillary bone, can all interfere with evaluation of the dentition. The interference of anatomic structures in evaluation of the dentition can be eliminated by evaluating the dentition in 2D MPR images. Rotating or cropping the 3D image can help increase visualization of these structures.

### Tooth Length and Width Measurements – CBCT

All tooth dimensions are expressed as means ±1 SD. The average lengths and widths of all teeth are presented in Table 1. The longest teeth were the maxillary and mandibular incisors, with lengths of 24.88 ± 2.31 and 23.24 ± 2.60 mm, respectively. The shortest teeth were the maxillary and mandibular third molar teeth, with the lengths of 7.31 ± 0.78 and 7.45 ± 0.95 mm, respectively. Within the mandible, the length of the teeth decreases in a caudal direction, meaning the premolar teeth are longer than the molar teeth. The thinnest teeth were the maxillary second incisor teeth (1.29 ± 0.26 mm) and the maxillary third molar teeth (1.04 ± 0.10 mm). The widest tooth was the mandibular third premolar tooth at 3.22 ± 0.28 mm. The remainder of the teeth were 2–3-mm wide.

**Table 1:** **Mean length and width of teeth in ten rabbits without dental disease**.

Tooth			Length (mm)	Width (mm)
Maxillary	Incisor	1	24.88 (±2.31)	2.04 (±0.18)
		2	9.51 (±1.46)	1.29 (±0.26)
	Premolar	2	12.06 (±0.98)	2.25 (±0.23)
		3	14.93 (±1.14)	2.17 (±0.24)
		4	15.29 (±1.18)	2.22 (±0.28)
	Molar	1	14.72 (±1.32)	2.19 (±0.27)
		2	13.26 (±1.42)	2.19 (±0.26)
		3	7.31 (±0.78)	1.04 (±0.10)
Mandibular	Incisor	1	23.24 (±2.60)	2.44 (±0.19)
	Premolar	3	16.19 (±0.71)	3.22 (±0.28)
		4	16.31 (±0.84)	2.65 (±0.19)
	Molar	1	15.75 (±0.74)	2.55 (±0.25)
		2	12.85 (±1.13)	2.59 (±0.25)
		3	7.45 (±0.95)	2.33 (±0.43)

*All values are means (±1 SD)*.

### Sagittal Images – CBCT and Conventional CT

The comparison of the sagittal tooth view and panoramic images when scoring the germinal center clarity, pulp cavity clarity, and tooth outline visibility can be viewed in Table 2. In general, the scores of the germinal centers were one score higher in CBCT than in conventional CT in both the tooth view and panoramic images. The right and left mandibular fourth premolar to the third molar teeth germinal centers were significantly (*P* < 0.01) more visible in CBCT than conventional CT, in both tooth view and panoramic images. There were no significant differences between the views of the right and left first incisor teeth (*P* = 0.22 and *P* = 0.11, respectively) and right and left first mandibular incisor teeth (*P* = 0.07 and *P* = 0.14, respectively) with regards to the germinal centers visibility in CBCT and conventional CT in tooth view.

**Table 2:** **Median difference between the score values of CBCT and conventional CT on sagittal tooth view, 3D reconstructed, and panoramic images**.

Tooth evaluated	GC tooth view	GC pano view	PC tooth view	PC pano view	TO tooth view	TO pano view
RmaxI1	0	1*	1*	1*	1*	2*
LmaxI1	0	0	1*	1*	1*	2*
RmaxI2	0.5*	1.5*	0	1*	1*	2*
LmaxI2	1*	0	0	0	1*	1*
RmaxP2	1*	1*	0	0	1*	1*
LmaxP2	1	1*	0	0	1*	1*
RmaxP3	1*	1*	1*	0.5*	0.5	1.5*
LmaxP3	1*	2*	1*	1*	1	1*
RmaxP4	1*	1*	2*	1*	1*	2*
LmaxP4	1*	1*	1*	1*	1*	2*
RmaxM1	1*	1*	2*	1*	1*	2*
LmaxM1	1*	1.5*	1*	1*	1*	2*
RmaxM2	1*	2*	1*	2*	1*	2*
LmaxM2	1*	1.5*	1*	1.5*	1*	2*
RmaxM3	0.5*	1*	0	0	3*	2*
LmaxM3	1*	0.5*	0	0	3*	1*
RmandI1	0	1*	1*	1*	1*	1*
LmandI1	0.5	1*	1*	1*	1*	1*
RmandP3	1*	1*	2*	3*	1*	1*
LmandP3	1*	1*	2*	3*	1*	1*
RmandP4	1*	1*	2*	3*	1*	2*
LmandP4	1*	1*	2*	3*	1*	2*
RmandM1	1*	1*	2*	2*	1*	2*
LmandM1	1*	1*	2*	2*	1*	2*
RmandM2	1*	1*	2*	1*	1*	1*
LmandM2	1*	1*	2*	1*	1*	1*
RmandM3	1*	1.5*	1*	2*	1*	3*
LmandM3	1*	2*	1*	2*	1*	3*

*All values are median differences*.

*GC, germinal center visibility; PC, pulp cavity visibility; TO, tooth outline visibility*.

*P-value <0.05.

There were no significant differences between the views of the left maxillary second incisor tooth pulp cavity in panoramic view (*P* = 0.18) and the right and left maxillary second incisor teeth pulp cavities in tooth view (*P* = 1, *P* = 1) on CBCT and conventional CT. The right maxillary second incisor tooth pulp cavity in panoramic view was significantly (*P* = 0.03) more visible in CBCT than conventional CT with a median difference in scoring of one. The right and left maxillary third premolar to the third molar pulp cavities had median differences of one-half to two with the CBCT images being significantly different than the conventional CT images in both imaging modalities (*P* < 0.05). There was a median difference of three, with all measurements being significant (*P* < 0.01) for the right and left mandibular third premolar and fourth premolar teeth pulp cavities on the panoramic images. In general, all the mandibular teeth pulp cavities were significantly (*P* < 0.05) more visible in CBCT than in conventional CT in both imaging modalities.

The outline of the right and left maxillary third molar was most visible on the tooth view, with a median difference of three and *P*-value <0.01. The median differences between CBCT and conventional CT were not significant for the right and left maxillary third premolar teeth outlines in tooth view. The right and left third molar teeth outlines were best viewed in panoramic images as the median difference between CBCT and conventional CT was three (*P* < 0.01). In the panoramic images, CBCT was always one score or more above the conventional CT images, and all differences were significant (*P* < 0.05).

### Multi-Planar Reconstruction Images – CBCT and Conventional CT

The results of evaluating the germinal center clarity, pulp cavity clarity, and periodontal ligament visibility in the transverse, dorsal, sagittal, and custom MPR images for both CBCT and conventional CT are shown in Table 3. In general, the CBCT image scores for the five selected teeth (right maxillary first incisor, right maxillary second premolar, right maxillary third molar, right mandibular fourth premolar, and right mandibular third molar tooth) were significantly higher (more clear images) than the conventional CT images. The exceptions were the right mandibular fourth premolar tooth germinal center clarity and the right maxillary first incisor tooth pulp cavity clarity, as these were just as visible on CBCT as conventional CT. Median differences for germinal center and pulp cavity scoring were small, ranging from one-half to two. The most dramatic finding was that the periodontal ligament was significantly (*P* < 0.01) more visible on CBCT than on conventional CT, with median differences ranging from one to three. The median difference of the periodontal ligament visibility for the right maxillary first incisor and right mandibular third molar teeth was three (*P* < 0.01). Overall, the germinal center, pulp cavity, and periodontal ligament of the shorter and thinner teeth (right maxillary third molar tooth, right mandibular third molar tooth) were all significantly more visible on CBCT than conventional CT.

**Table 3:** **Median score, range, and median difference of CBCT and conventional CT transverse, sagittal, dorsal, and custom MPR images for the germinal center, pulp cavity, and periodontal ligament visibility**.

Category	Tooth evaluated	CBCT median score (range)	Conventional CT median score (range)	Median difference
Germinal center visibility	RmaxI1	4 (4–4)	3 (3–4)	1*
	RmaxP2	4 (4–4)	3 (3–3)	1*
	RmaxM3	3 (3–3)	1 (1–2)	2*
	RmandP4	4 (4–4)	4 (4–4)	0
	RmandM3	4 (4–4)	2 (2–3)	2*
Pulp cavity visibility	RmaxI1	4 (4–4)	4 (4–4)	0
	RmaxP2	4 (3–3)	3 (3–3)	1*
	RmaxM3	2 (1–2)	1 (1–2)	1*
	RmandP4	4 (4–4)	3.5 (3–4)	0.5*
	RmandM3	4 (4–4)	2 (1–3)	2*
Periodontal ligament visibility	RmaxI1	4 (4–4)	1 (1–1)	3*
	RmaxP2	4 (4–4)	2 (1–2)	2*
	RmaxM3	3.5 (3–4)	1 (1–1)	2.5*
	RmandP4	4 (4–4)	3 (3–3)	1*
	RmandM3	4 (3–4)	1 (1–1)	3*

*P-value <0.05.

## Discussion

### Gross Evaluation and Anatomy

The present study demonstrates that CBCT images are superior to conventional CT images when evaluating the dentition and osseous maxillofacial structures of the rabbit. We were able to complete our first aim and identify and label the anatomic structures of the maxillofacial region as well as measure the lengths and widths of the teeth using CBCT. Secondly, we were also able to demonstrate the differences in visibility of various dental structures, including the germinal center, pulp cavity, and periodontal ligament by CBCT and conventional CT.

Cone-beam CT allows better detection of subtle anatomic changes compared to conventional CT when imaging the dentition and bony maxillofacial structures in the rabbit. Despite the fact that the resolution of analog or digital conventional radiology is superior to CT scans, viewing images of the patient in sagittal, dorsal, transverse, MPR, panoramic, and volume renderings offers clinically important advantages (21). Traditional radiography was not compared to CBCT and conventional CT in this study, because the fine details of the dentition are difficult to discern due to the superimposition of anatomic structures on skull radiographs. In 2011, Van Caelenberg et al. compared radiography and conventional CT in order to identify changes in skulls of four rabbits with dental disease. It was concluded that compared to radiography, the CT images provided more details about the extent of osseous changes and dental pathology, which is important for establishing a more precise prognosis and treatment plan (17).

A disadvantage of clinical use of CBCT in animals, compared with conventional CT, includes the maximum size of objects that can be scanned. The NewTom 5G CBCT produces high-resolution images with six programed fields of view ranging from 6 cm width by 6 cm length to 18 cm width by 16 cm length. The finest spatial resolution is limited to the smallest FOV, which is not sufficient to evaluate the entire skull of a rabbit. Some systems or software packages may allow more than one CBCT scan to be acquired and digitally “stitched” together to seamlessly evaluate a larger region. We found that acquisition setting of CBCT images used in this study allowed for imaging of the entire rabbit skull with excellent resolution of dentition and maxillofacial structures.

Due to the obvious differences in image quality between the conventional CT and CBCT, the comparison of the images could not be blinded. Part of the difference observed between CBCT and conventional CT is due to differences in slice thickness between the two CT modalities. It is important to emphasize that the CBCT hardware has been optimized to provide high-resolution dental and maxillofacial images, whereas conventional CT is capable of evaluating both the bone and soft tissues. Conventional CT provides superior images of soft tissues due to having a higher contrast:noise ratio. Selection of the proper method of advanced diagnostic imaging should be based on hardware availability, history, clinical signs, and physical examination findings. If a purely dental disease is suspected, CBCT should be the method of choice for rabbits. If neoplasia is suspected or a soft-tissue disease is present that may not be related to dental disease, then conventional CT should be selected.

### Tooth Length and Width Measurements

To our knowledge, the present study is the first to report the average tooth length of rabbit teeth not affected by dental disease. Knowing the average length of the tooth or being able to measure it for an individual patient may help veterinarians understand how much tooth to expect to remove when performing extractions. If the tooth is shorter than expected during extraction, fracture of the reserve crown and the presence of a tooth remnant may have occurred. Alternatively, some teeth may be shorter due to inflammatory root resorption. Unfortunately, intraoperative imaging, such as a repeat CBCT or dental radiographs to determine the presence of a tooth remnant, may not be feasible in rabbits due to increased anesthesia time, increased cost to the client, and the challenge of obtaining intraoral dental radiographs in this species.

Tooth length may vary with breed and skull conformation. The New Zealand white rabbits used in this study have a longer skull and weigh more than rabbits commonly afflicted by dental disease, such as the Netherland dwarf, mini lop, and Holland lop (30). The data collected regarding tooth length can be used in future studies for comparison to both normal and diseased rabbits of different or similar breeds.

There may be some error when the tooth length measurements were collected, as the lengths were measured from two-dimensional sagittal images. In normal teeth, the error in measurement may be negligible. The right and left mandibular third premolar to the third molar teeth are maintained within the mandibular bone and do not protrude medially or laterally on rabbits without dental disease. The germinal centers of the right and left maxillary second premolar and second molar teeth slightly curve laterally on a normal rabbit but may be significantly curved in multiple directions in a rabbit with apical elongation. Tooth lengths were not measured on transverse images, as the entirety of the tooth was not always visible in a single slice, and the measuring tools available in the software program were limited depending on which section of the software was being used.

In addition to being the first study to report the average length of rabbit teeth, this is the first study to report the average width of the teeth. The clinical significance is that in a conventional CT with 0.625-mm slice thickness, there will be one to two CT imaging slices collected for diagnostic interpretation on these thin teeth, which means that the majority of the tooth is being skipped in the imaging process. In a CBCT with the smallest slice thickness of 0.15 mm, six to eight CT imaging slices will be collected over this same distance, allowing for better evaluation of even the thinnest teeth. In this study, the smallest slice thickness of the CBCT was limited to 0.2 mm due to the scanning window required. This would allow for five to six CT imaging slices to be collected for the second maxillary incisor and third maxillary molar teeth. The widest tooth was the mandibular third premolar tooth at 3.22 ± 0.28 mm. It makes sense that the mandibular third premolar would be the widest tooth as there are six maxillary premolars and molars and only five mandibular premolars and molars. All of the maxillary second premolar tooth and part of the maxillary third premolar tooth occlude on the mandibular third premolar tooth.

The evaluation of the tooth width with respect to CT slice thickness does not apply to the maxillary and mandibular first incisor teeth, since these teeth are shaped in an arc within the maxilla and mandible. As both the CT scans collect and report DICOM images in transversely oriented slices, the images are not being collected in parallel to the axis of the tooth.

### Sagittal Images – CBCT and Conventional CT

In the clinical setting, it is relatively rapid to evaluate the dentition from a CT scan by creating the tooth view 3D reconstruction as well as the panoramic image. These images will provide the operator with a “global feel” of the dental disorders occurring in the mouth and their relationship to one another. In contrast, scrolling back-and-forth through the sagittal, transverse, dorsal, and custom MPR images for each of the 28 teeth can be time consuming but can clarify any questions that might arise when viewing the two-dimensional sagittal images or 3D reconstructions. Although 3D reconstructions are valuable for evaluating structures with complex geometries, it is important to keep in mind that internal features can only be fully evaluated on 2D images. Additionally, evaluation of the soft tissues using 2D images with an appropriate window is critical, as soft-tissue disorders such as abscesses and exophthalmos often accompany dental disease in rabbits.

The germinal centers appear as lucencies at the periapical region of the teeth; therefore, they might be confused as periapical lucencies (i.e., abscesses or granulomas). It is important to clearly identify the germinal centers and understand their boundaries so that pathology can be identified when present. On the sagittal CBCT images, the germinal centers were visible in both the tooth view and panoramic images. In general, the mandibular premolar and molar germinal centers are more easily viewed due to the lack of overlap of adjacent anatomic structures and due to the germinal centers diverging from one another. In the maxilla, the zygomatic arch and the closely spaced teeth make visualization of the germinal center difficult on tooth view and panoramic images; therefore, the zygomatic arch was cropped-out of these images before evaluation. The germinal centers of the maxillary second premolar to third molar teeth were often not clear, as the maxillary alveolar bone separating the teeth from the retrobulbar space is thin, so there is no striking contrast between the alveolar bone and the germinal center as one would see in the mandibular premolar and molar teeth.

When comparing the CBCT and conventional CT panoramic and tooth views, the pulp cavity becomes completely obscured on the conventional CT for all of the maxillary teeth except for the first incisor teeth. For conventional CT, the mandibular first and second molar teeth have pulp cavities that can still be assessed on panoramic view but not on tooth view; otherwise the remaining pulp cavities of the premolars and molars cannot be assessed in the two-dimensional images.

The periodontal ligament was not visualized well on the two-dimensional sagittal tooth and panoramic images; therefore, the ability to discern one tooth from another by scoring the outline of the tooth was used for evaluation. On tooth view, the mandibular fourth premolar and first molar teeth all blur together where they are nearest to one another coronally. Decreasing the opacity of the tooth view control allowed for better visualization of the outline of the tooth in the 3D volume render. The maxillary second premolar to the fourth premolar teeth was often 25–50% obscured by the palatine process of the maxillary bone and zygomatic arch. The outline of the maxillary third molar teeth was most visible on the tooth view with the CBCT image being by far superior to the conventional CT image.

### Multi-Planar Reconstruction Images – CBCT and Conventional CT

Overall, it was determined that, although faster, evaluating the germinal center, pulp cavity, and tooth outline visibility was not ideal on the tooth view and panoramic images. To prevent the superimposition of anatomic structures and to increase the diagnostic yield of the images, the germinal center, pulp cavity, and periodontal ligament were viewed in transverse, sagittal, dorsal, and custom MPR views.

In general, the CBCT image scores for the five selected teeth (right maxillary first incisor, right maxillary second premolar, right maxillary third molar, right mandibular fourth premolar, and right mandibular third molar tooth) were significantly better than the conventional CT images. The germinal center, pulp cavity, and periodontal ligament of the shorter and thinner teeth (right maxillary third molar and right mandibular third molar teeth) were all significantly more visible on CBCT than conventional CT. An important finding was that the periodontal ligament was significantly (*P* < 0.01) more visible on CBCT than on conventional CT with median differences ranging from one to three. The median difference of the periodontal ligament visibility for the right maxillary first incisor and right mandibular third molar teeth was three (*P* < 0.01). This is the first study to evaluate the clarity of the periodontal ligament on any imaging modality. Ability to see the periodontal ligament with such detail may allow the earlier detection and treatment of periodontal disease in rabbits, thereby slowing progression.

In conclusion, our hypothesis that CBCT images are superior to conventional CT when imaging the dentition and bony maxillofacial structures of the rabbit was supported by our findings. We were able to demonstrate the differences in visibility of various dental structures, including the germinal center, pulp cavity, and periodontal ligament. With the improvement in imaging technology and the availability of specialized software, these dental structures can now be better analyzed for the presence of pathology. This study provides the baseline for introducing CBCT for evaluating small animal maxillofacial features and dentition.

## Ethics Statement

The study was approved by the Clinical Trial Review Board – University of California – Davis.

## Author Contributions

GR: study concept and design; acquisition of data; analysis and interpretation of data; drafting of the manuscript. BA and FV: study concept and design; analysis and interpretation of data; critical revision of the manuscript for important intellectual content; administrative, technical, or material support; study supervision; obtaining funding. DC: analysis and interpretation of data; critical revision of the manuscript for important intellectual content. DH: critical revision of the manuscript for important intellectual content. PK: statistical analysis. AZ: acquisition of data; drafting of the manuscript. Dr. FV had full access to all of the data in the study and takes responsibility for the integrity of the data and the accuracy of the data analysis.

## Conflict of Interest Statement

The authors declare that the research was conducted in the absence of any commercial or financial relationships that could be construed as a potential conflict of interest.
